# Food and macronutrient intake of elite Ethiopian distance runners

**DOI:** 10.1186/1550-2783-8-7

**Published:** 2011-05-19

**Authors:** Lukas Y Beis, Lena Willkomm, Ramzy Ross, Zeru Bekele, Bezabhe Wolde, Barry Fudge, Yannis P Pitsiladis

**Affiliations:** 1 College of Medicine, Veterinary & Life Sciences. Institute of Cardiovascular & Medical Sciences, University of Glasgow, UK; 2 German Sports University Cologne, Department of Molecular and Cellular Sports Medicine, Cologne, Germany; 3 Department of Physical Education and Sport Science, Addis Ababa University, Addis Ababa, Ethiopia; 4 English Institute of Sport, Loughborough University, Loughborough, UK

## Abstract

**Background:**

Explanations for the phenomenal success of East African distance runners include unique dietary practices. The aim of the present study was to assess the food and macronutrient intake of elite Ethiopian distance runners during a period of high intensity exercise training at altitude and prior to major competition.

**Methods:**

The dietary intake of 10 highly-trained Ethiopian long distance runners, living and training at high altitude (approximately 2400 m above sea level) was assessed during a 7 day period of intense training prior to competition using the standard weighed intake method. Training was also assessed using an activity/training diary.

**Results:**

Body mass was stable (i.e., was well maintained) over the assessment period (pre: 56.7 ± 4.3 kg vs. post: 56.6 ± 4.2 kg, *P *= 0.54; mean ± SD). The diet comprised of 13375 ± 1378 kJ and was high in carbohydrate (64.3 ± 2.6%, 545 ± 49 g, 9.7 ± 0.9 g/kg). Fat and protein intake was 23.3 ± 2.1% (83 ± 14 g) and 12.4 ± 0.6% (99 ± 13 g, 1.8 ± 0.2 g/kg), respectively. Fluid intake comprised mainly of water (1751 ± 583 mL), while no fluids were consumed before or during training with only modest amounts being consumed following training.

**Conclusions:**

Similar to previous studies in elite Kenyan distance runners, the diet of these elite Ethiopian distance runners met most recommendations of endurance athletes for macronutrient intake but not for fluid intake.

## Background

The International Association of Athletic Federations (IAAF) Consensus Statement on Nutrition for athletics published in 2007 states: "Well chosen foods will help athletes train hard, reduce risk of illness and injury, and achieve performance goals, regardless of the diversity of events, environments, nationality and level of competitors." [1]. Specific nutritional recommendations for optimal performance, particularly for endurance athletes, include a daily carbohydrate (CHO) intake ranging from 6 to 10 g/kg body mass (BM) considered essential for replacing liver and muscle glycogen stores [2]. A significant protein intake ranging between 1.2 to 1.7 g/kg BM per day is required for optimal health and performance of endurance athletes [2]. Studies examining protein intake in athletes have shown an increased requirement for protein in endurance trained athletes [3-5] as opposed to healthy adult males (i.e., 0.8 g/kg) due to increased amino acid oxidation during exercise and for growth and repair of muscle tissue [6]. Maintenance of normal body water during strenuous training and minimising the level of dehydration (i.e., preventing a BM loss of > 2%) during endurance exercise achieved by consuming fluids at a rate of 0.4 to 0.8 L/h *ad libitum *is now recommended [7]. More studies that employ truly world class athletes are required in order to assess and possibly improve the applicability of the current recommendations to elite athletes. Therefore, there is an urgent need for novel data that can be obtained from some of the best athletes in the world.

Ever since Abebe Bekele became the first black African gold medalist in winning the marathon at the Rome Olympics in 1960, scientists have tried to explain the phenomenal success of east African distance runners in international athletics [8-11]. Notably, middle- and long-distance runners from Ethiopia and Kenya hold over 90% of both all-time world records as well as the current top-10 positions in world event rankings [12]. Possible explanations have been proposed including genetic factors [13,14], environmental conditions [9,15] and near optimal dietary practices [9,16,17]. However, the east African running phenomenon still remains largely unexplained. While a significant number of studies have investigated putative factors influencing the east African running phenomenon, only five studies have assessed the dietary practices of elite east African runners and all have involved Kenyan athletes [8,9,16-18]. The first of these studies, Mukeshi and Thairu [17] estimated the energy intake (EI) of male, long distance Kenyan runners through a combination of questionnaires and direct observation. Remarkably low EI (9790 kJ/d on average) was reported, while the average CHO intake was 441 g (8.1 g/kg of BM per day) or 75% of total EI (TEI). However, in the subsequent studies [8,9,16,18], substantially higher estimates of EI were noted in comparison to the initial data. For example, Christensen et al. [16] reported an average EI of 13210 kJ/d, while the consumption of CHO was 476 g (8.7 g/kg BM, 71% of TEI). Similarly, Onywera et al. [9] reported an average EI of 12486 kJ/d (CHO 607 g, 10.4 g/kg BM and 76.5% TEI), while estimated EI in two studies by Fudge and colleagues were 13241 kJ/d (CHO 552 g, 9.8 g/kg BM and 71% TEI) [18] and 12300 kJ/d (CHO 580 g, 9.8 g/kg BM, 79% TEI) [8], respectively. These dietary studies focused primarily on athletes from the Kalenjin tribe of Kenya; a fairly distinct Kenyan ethnic group living at high altitudes, noted for producing athletes of great endurance. For example, the Kalenjin tribe has less than 0.1% of the world's population, yet members of this tribe have achieved nearly 50 athletic Olympic medals.

Ethiopian athletes boast a recent success record in international distance running second only to Kenya. As is the case in Kenya, successful Ethiopian athletes come predominantly from one localized ethnic group in the Ethiopian region of Arsi [14]. The Arsi region of Ethiopia is situated at high altitude and contains roughly 5% of the Ethiopian population whilst accounting for 14 of the 23 distance runners selected for the country's 2008 Olympic team. Historically there has been very little cultural exchange or indeed biological intermingling between Kenyans [10] and Ethiopians, therefore very distinct dietary habits can be found in the two countries, even between tribes within each country. Consequently and given the absence of dietary data for Ethiopian athletes, the main aim of the present investigation was to assess the dietary practices of elite Ethiopian endurance runners to elite Kenyan athletes during an important training period, as well as to the current recommendations for endurance athletes. This investigation also aimed to provide a rare insight into the lifestyle and training practices of some of the most successful endurance runners in the world prior to major competitions.

## Methods

### Subjects

Ten highly-trained (8 male, 2 female) Ethiopian distance runners gave their written informed consent to take part in the present study which was approved by the local ethics committee (Research Ethics Committee, Department of Physical Education and Sport Science, Addis Ababa University, Addis Ababa, Ethiopia) and was performed according to the code of ethics of the World Medical Association (Declaration of Helsinki). Subjects were highly trained (best marathon time: 2:13:55 ± 0:01:42; mean ± SD; Table 1) and in excellent condition (trained twice daily) while preparing for major competitions (e.g., 2008 Beijing Olympic Games, 2008 Berlin marathon). Athletes recruited were managed by Global Sports Communication http://www.globalsportscommunication.nl/; arguably the most accomplished of all the track and field athlete management organizations specializing in middle- and long-distance running events. Athletes living and training at the training camp under the management of Global Sports Communication all follow very similar training practices. Athletes residing at the Global training camp included world record holders, medalists at major championships such as the Olympic Games, World Championships and major city marathons like the London Marathon. The present study was conducted during the period when some of the athletes were preparing for the 2008 Beijing Olympics. The physical characteristics of the athletes included in the present study were measured according to the 2006 ISAK procedures [19] and are presented in Table 1.

**Table 1:** Physical characteristics of the Ethiopian runners

Subject (no)	Age (y)	Height (m)	Start BM (kg)	End BM (kg)	Change BM (%)	Change BM (kg)	BT (M)	BT (F)
1	23	1.72	58.7	58.7	0.0	0.0	2:12:00	
2	21	1.78	62.4	61.5	1.4	-0.9	2:12:00	
3	22	1.72	59.8	59.9	-0.1	0.1	2:13:15	
4(F)	19	1.75	57.3	57.4	-0.2	0.1		2:35:03
5(F)	19	1.61	48.8	48.3	1.0	-0.5		2:30:15
6	23	1.73	57.7	58.5	-1.4	0.8	2:15:15	
7	27	1.81	53.5	53.3	0.4	-0.2	2:14:10	
8	20	1.76	61.7	61.0	1.1	-0.7	2:12:35	
9	23	1.73	53.4	53.6	-0.4	0.2	2:15:45	
10	23	1.65	53.3	53.4	-0.2	0.1	2:16:17	
Average	22	1.73	56.7	56.6	0.2	-0.1	2:13:56	
SD	2	0.06	4.3	4.2	0.8	0.5	0:01:42	

* Note: M, male; F, female; BM, body mass; BT, best marathon time.

### Study design

The dietary intake of the athletes was assessed in the month of July (ambient temperature: 12 to 21°C) during a period of intense training prior to major competition using 7 day weighed dietary records [20]. The dietary intake of the athletes was directly observed, weighted and recorded. All athletes competed in endurance running events ranging from 10 km to the marathon and lived in a single training camp (Global Sports training camp Addis Ababa - Kotebe, 8° 58' 0 N, 38° 49' 60 E) which was based at high altitude (~2400 m above sea level). During the 7 days, subjects followed their habitual eating/drinking pattern, as was confirmed by the manager/coach of the training camp. Training was assessed using a training diary which included the type, intensity and duration of exercise training. The training diary, in combination with direct observation, was used to estimate energy expenditure (EE) (Table 2). Briefly, total EE was estimated from the duration and intensity of each activity, using physical activity ratios (PAR) [21]. The energy cost was expressed as a multiple of basal metabolic rate (BMR). In the current study, BMR was estimated using the Schofield equations [22]. It should be noted that the training intensity and EE data has been generated in the present study using indirect methods [21]. Nevertheless, the results of these indirect methods are reported in order for the results of the current study to be directly comparable to the data generated in previous studies using similar methods [9].

**Table 2:** Estimated daily energy expenditure according to Physical Activity Ratio

		Duration (h)		Energy cost (PAR)	
		
	**PAR**^**a**^	MEAN	SD	MEAN	SD
Sleeping	0.9	9.0	0.8	8.1	0.7
Relaxing^b^	1.0	5.7	0.5	5.7	0.5
Miscellaneous activity^c^	1.5	6.7	0.0	10.1	0.0
Light exercise^d ^(Home activities)	3.0	0.5	0.1	1.4	0.2
Slow pace running	10.0	0.1	0.2	1.5	1.6
Moderate pace running	14.0	0.9	0.3	13.1	3.7
Fast pace running	18.0	0.7	0.2	12.2	4.0

Total		24	0.6	52.1	3.3

* Note: SD, standard deviation. ^a^Physical activity ratio (PAR) is the energy expenditure expressed in relation to basal metabolic rate (BMR) (i.e., BMR × 1.0). ^b^Watching TV, sitting quietly. ^c^Eating, socializing. ^d^Home activities.

The subjects weighed and recorded all food and drink consumed using individual weighing scales accurate to 1 g (Salter Housewares LTD, England). All food was weighed before and after cooking. The cooking method was also described and recorded. The participants were also required to use the weighing scales when they were away from the training camp and to disclose any extra food intake during the hours when direct observation was not possible. Details on how to report food and fluids consumed were given to each subject in English and in their local dialect (i.e., Oromo or Amharic). Total water intake was assessed by combining the reported dietary intake of water with the estimated metabolic water value as previously described and conducted in elite Kenyan athletes [8,18]. Metabolic water was determined by multiplying the measured EE by the fraction of energy in the diet obtained from CHO, protein and fat (data derived from the 7 day nutritional records). This value was then multiplied by water obtained from CHO, protein and fat oxidation (0.60, 0.41 and 1.07 mL water/g, respectively) [23]. To improve the quality of the collected data and to avoid any problems or under reporting of food or fluids consumed, one of the researchers resided at the camp for the entire assessment/observational period. Meals were prepared whilst athletes trained and served at the same times every day: Breakfast was at 09:30, after the morning training session, lunch at 13:30 and dinner at 19:30. On some occasions, athletes also had an afternoon snack which was served at 16:00. Nude BM was measured on the first day of the assessment period (as well as for two days prior to the start of the assessment period to ensure a representative baseline) and at the end of the 7 day period, before the consumption of any food or drink. The weighed dietary intake data was used to determine EI and diet composition using a computerised version of the food composition tables of McCance and Widdowson as revised by Holland et al. [24]. However, for foods more specifically consumed by Ethiopians, food tables published by the Ethiopian Ministry of Health of Ethiopia were used [25]. No samples were retained for further analysis due to local regulations. Food labels were also collected where possible, mainly for imported foods.

### Statistical analysis

Data was expressed as the mean ± standard deviation, as appropriate following a test for the normality of distribution. Paired t-tests were used to compare EI vs. EE and starting BM vs. final BM. Statistical significance was declared when *P *< 0.05. All statistical analysis was completed using the software package SPSS, version 15.0 (SPSS, Inc., Chicago, IL, USA).

## Results

Training typically consisted of two sessions per day. The morning run (normally at 07.00) took place before breakfast and included a session at moderate or fast pace (16-20 km/hr) for 10 to 20 km depending on the instructions given by the coach and/or weather conditions. The afternoon session, prior to dinner (17.00), was typically an easy run over 6 to 10 km at a slower pace (10-15 km/hr), unless morning weather conditions had been adverse. If this was the case, athletes reversed their sessions. Warming up periods were 15 min and cooling down periods were more than 20 min. Warm up and cool down consisted of standard stretching exercises and athletes carried out most of their sessions as a group. In some instances, some athletes trained alone. Athletes completed high intensity interval training sessions 2-3 times per week and one 20-25 km run at near race speed for each athlete. Recovery time between training sessions was spent at the camp sleeping, eating, socialising, watching television or washing their clothes. Some athletes went home on weekends and completed individual training runs as advised by their coach/manager. The EE of the athletes as estimated using PAR is shown in Table 2.

**Figure 1:**
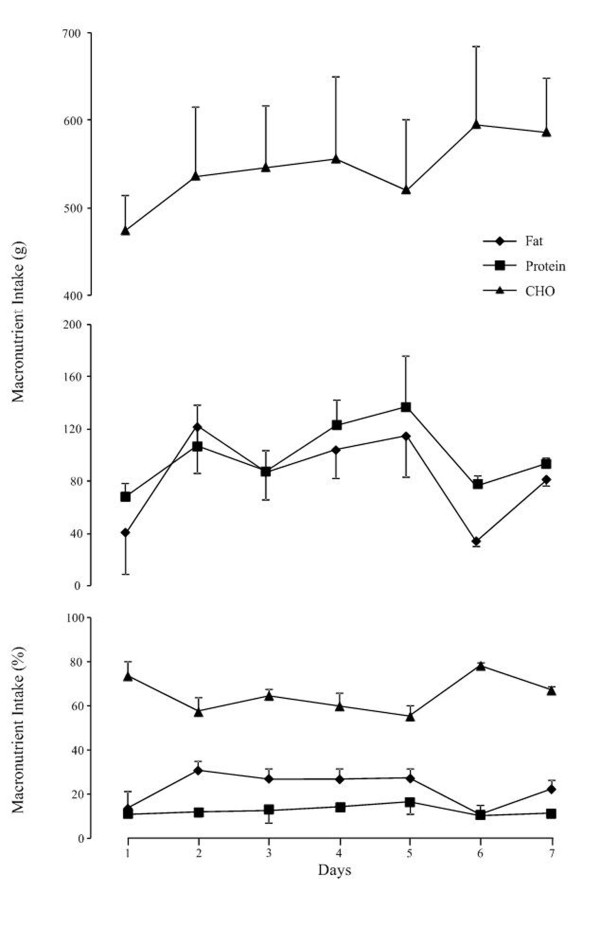
**Macronutrient intake (g and percent intake) (mean ± standard deviation) over the 7 day period**.

**Figure 2:**
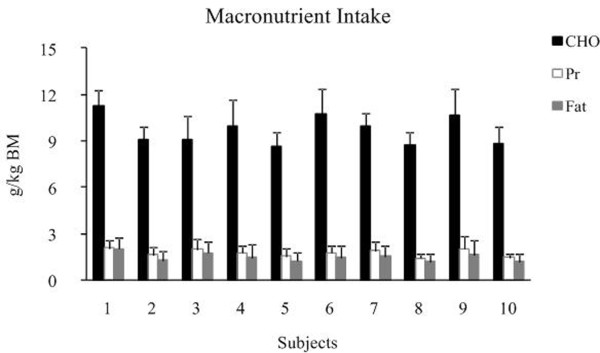
**Individual ranges of macronutrient intake (average for the 7 day period)**.

Estimated EI over the 7 day assessment period (13375 ± 1378 kJ) was matched by estimated EE (13670 ± 862 kJ; *P *= 0.69). BMR was estimated at 6292 ± 565 kJ per day. The BM remained stable over the 3 days prior the assessment period (pre: 56.6 ± 4.1 kg vs. post: 56.7 ± 4.3 kg; *P *= 0.58). The athletes' BM (pre: 56.7 ± 4.3 kg vs. post: 56.6 ± 4.2 kg; *P *= 0.54) remained stable over the 7 days (Table 1). The diet consisted mainly of vegetable sources (approximately 88%) with only a small portion of meat (approximately 12%) (Table 3). Breakfast consisted typically of milk, porridge, omelet and bread. Lunch comprised mainly of vegetable sources such as pasta, rice and lentils, while meat was served only twice a week and dinner was similar to lunch. Food portions were chosen by the subjects themselves (i.e., *ad libitum*), as no advice or guidelines were given. Furthermore, two of the athletes consumed commercially available nutritional supplements (i.e., 100 g of the supplement consisted of 95.1 g CHO of which sugars 59.7 g, L-Glutamine 250 mg, L-Leucine 110 g, L-Valine 100 g, L-Isoleucine 70 mg, and Sodium 0.9 g). As for fluid intake, subjects consumed water with modest amounts of tea, milk, orange juice and a local drink called Besso, a mixture of barley and water. The diet was high in CHO intake (64.3 ± 2.6%, 545 ± 49 g, 9.7 ± 0.9 g/kg per day (Figure 1, Figure 2). The fat intake of the diet was 23.3 ± 2.1% and 83 ± 14 g daily (Figure 1, Figure 2). Protein intake was 12.4 ± 0.6%, 1.8 ± 0.2 g/kg and 99 ± 13 g per day (Figure 1, Figure 2) of which 76% was derived from vegetable sources (Table 3). Daily fluid intake consisted mainly of water (1751 ± 583 mL; 55.4% of the total water intake), while the athletes did not consume any fluids before or during their training sessions. Other sources of daily fluid intake were water consumed as moisture in food (950 ± 60 mL; 29.9%) and metabolic water produced as a result of the oxidation of CHO, protein, and fat (470 ± 28 mL; 14.8%) which resulted in a mean total daily fluid intake of 3.2 ± 0.6 L/day.

**Table 3:** Food Sources as a percentage of daily intake of each macronutrient

Food Sources (%)	Energy (kcal)	CHO (g)	Fat (g)	Protein (g)
Porridge	4.5	5.5	2.1	3.0
Bread	15.2	18.7	4.7	17.5
Pasta	10.0	12.0	3.1	13.4
Rice	5.0	6.5	1.8	2.8
Injera	20.8	27.3	4.8	16.5
Meat	5.3	0.1	16.1	11.9
Lentils	2.4	1.8	3.6	3.5
Sugar^a^	3.5	5.4	0.0	0.0
Eggs	1.5	0.1	3.9	4.0
Milk	1.3	0.6	3.1	2.1
Vegetable Oil	10.2	0.0	43.5	0.0
Chick Peas	1.0	0.9	0.6	1.9
Shiro	2.1	1.5	2.4	4.7
Total	83	85	90	84
Other^b^	17	15	10	16
				
Animal source	12	1	27	24
Vegetable source	88	99	73	76
				
Mean	3194	545	83	99
SD	329	49	14	13

*Note. SD, standard deviation. ^a^Sugar consumed in tea and porridge. ^b^Food sources that contribute less than 1%.

## Discussion

The findings of the present study indicate that Ethiopian endurance runners whilst meeting dietary recommendations for endurance athletes for macronutrient intake did not do likewise as far as fluid intake was concerned. In the present study, the dietary intake data was used to estimate the EI, while the EE and BM data were interpreted in the context of energy balance and in order to assess under eating. Total average EI was 13375 ± 1378 kJ and is in agreement with previous studies [8,9,16,18] (~ 12809 kJ/d on average). In the first of these studies conducted in Kenyan athletes, Mukeshi and Thairu [17] estimated the EI of male, long distance Kenyan runners through a combination of questionnaires and direct observation and reported remarkably low EI (9790 kJ/d on average). However, in subsequent studies [8,9,16,18], substantially higher estimates of EI were reported in comparison to the initial data. For example, Christensen et al. [16] reported an average EI of 13210 kJ/d. Similarly, Onywera et al. [9] reported an average EI of 12486 kJ/d, while estimated EI in two studies by Fudge and colleagues were 13241 kJ/d [18] and 12300 kJ/d [8]. A finding common to most of the aforementioned studies was the lower EI compared to EE and therefore indicative of negative energy balance before major competition [9,18]. It is well acknowledged that training at high altitude can impact negatively on energy balance [26], most likely due to a reduction in EI brought about by a loss of appetite [27]. However, in contrast to previous studies in Kenyan runners [9,18], Ethiopian runners recruited in this study met their energy needs (EI did not differ from EE) and consequently maintained their BM (pre assessment period BM: 56.7 ± 4.3 kg vs. post: 56.6 ± 4.2 kg). This is consistent with recent guidelines by the American College of Sport Medicine that advocate that differences between EI and EE could compromise performance and negate the benefits of training [2].

Macronutrient intake of Ethiopian long distance runners fulfilled recent recommendations [2]. CHO intake was 64.3% (9.7 g/kg per day) and the daily CHO intake was 545 ± 49 g (Figure 1), while recommendations for male and female athletes range between 6 to 10 g/kg of BM per day [2]. These results are also in agreement with previous studies [8,9,16-18] when the daily amount of CHO was well above 65% of TEI, ranging from 8.1 to 10.4 g/kg BM and within the current recommendations [2]. Protein intake was 12.4% of TEI (Figure 1) (1.76 g/kg BM per day with a daily intake of 99 ± 13 g) of which 76% was delivered from vegetable sources (Table 3) and well within the current recommendations for endurance athletes (1.2 to 1.7 g/kg BM per day) [2]. This is also in agreement with the literature [8,9,16,18] where daily protein intake ranged from 1.3 to 2.2 g/kg BM. Adequate protein and fat intake are also vital for optimal health and performance of long distance runners. Sufficient dietary protein will provide essential amino acids and maintain the nitrogen balance for building and repair of muscle tissue after intense endurance training [2]. Furthermore, having achieved the recommended amounts of CHO and protein, this would have resulted in a sufficiently high intake of fat to ensure an important source of fat soluble vitamins and essential fatty acids [2,28]. Hence, the fat intake of distance runners especially from developing countries should not be restricted further as there would be no performance benefit in consuming less fat than that observed in the current study (23.3% TEI). Rodriguez et al. [2] reported that there are no advantages in consuming a diet with less than 15% of energy from fat compared with 20 to 25% of TEI. Although, the values from the present study (23.3% TEI, Figure 1) for fat intake are in agreement with the guidelines [2], they were somewhat higher in comparison to values (6.6 to 17.4% of TEI) observed in previous studies [8,9,16-18]. Moreover, the fact that vegetable sources accounted for approximately 88% of TEI (Table 3) concurs with other published dietary studies for low income countries [16,17,29] and contrasts with that for developed countries [30-32]. For example, the CHO intake of elite distance runners in the United States [31], the Netherlands [32] and Australia [30] was 49%, 50% and 52% respectively, as a result of a more varied diet.

Optimizing fluid replenishment is fundamental during exercise. Correct fluid replacement practices are especially crucial in endurance events lasting longer than an hour where the participating athlete might have not consumed adequate food or fluid before exercise or in cases where the athlete is exercising in an extreme environment (heat, cold, or high altitude) [2]. It is perhaps surprising that in the present study, the Ethiopian endurance athletes taking part in prolonged intense exercise and/or extreme conditions, did not fulfil the current recommendations for fluid intake [7]. In fact, the athletes consumed approximately 1.75 L/day of fluids which comprised mainly of water and athletes in general did not consume water before or during training; in some occasions small amounts of water was consumed following training. This finding is in line with previous findings [8,9,18]. Onywera and colleagues [9] reported a modest fluid consumption (2.3 L/d). Additionally, similar fluid intake (1.8 L/d) was observed by Fudge et al. [18] and in a subsequent study by the same group (2.3 L/d) [8]. These studies collectively show that these elite athletes do not consume any fluids before or during training, while modest amounts of fluids are consumed after training and only by a small number of runners [8,9,18]. According to current recommendations, the amounts of fluid consumed (as dietary water intake) in the present study would be inadequate to maintain athletes' hydration status [7]. Nevertheless, when total water intake (i.e., dietary water intake and metabolic water) is considered, Ethiopian athletes are found to be well hydrated during the day due to the high quantity of water in their staple foods (e.g., injera). Furthermore, although fluid consumption in the present study was less than recommended [7], the daily total *ad libitum *water intake (0.23 ± 0.04 L/MJ) was consistent with guidelines from the US National Research Council [33]. These guidelines suggest 1 mL of water per kcal (0.24 L/MJ) of EE for adults under average conditions of EE and environmental exposure with the rare exception of instructing the consumption of 1.5 mL/kcal (0.36 L/MJ) in cases of higher levels of physical activity, sweating and solute load. Additionally, the total water intake in the current study (3.2 L) is in accordance with optimal kidney function and urine output maintenance at high altitude (i.e., 3-4 L/day) [2]. This is also in agreement with the existing literature [8,9,18] where elite Kenyan distance runners maintained their hydration status due to the consumption of foods with a high quantity of water (e.g., ugali) [9]. On the other hand, fluid intake recommendations as set by the ACSM guidelines indicate that athletes should consume 5-7 mL/kg of BM of fluids at least 4 hours prior to the exercise session aiming to start the physical activity euhydrated with normal plasma electrolyte levels [7]. Nevertheless, evidence to support this recommendation is equivocal at this point. It is important to note that mild dehydration may actually be an advantage as, theoretically, it will lower the energy cost of running at the same relative intensity [34,35].

## Conclusions

As previously found in elite Kenyan endurance runners, elite Ethiopian runners met dietary recommendations for endurance athletes for macronutrient intake but not for fluid intake. Nevertheless, it remains unclear how these differences in dietary patterns with regard to fluid consumption, before major competitions, impact on their performance.

## Competing interests

The authors declare that they have no competing interests.

## Authors' contributions

LYB was the primary author of the manuscript. LW was involved in subject recruitment, data collection and helped to draft the manuscript. RR was involved in subject recruitment, data collection and helped to draft the manuscript. ZB was involved in subject recruitment, data collection and editing the manuscript. BW was involved in subject recruitment, data collection and editing the manuscript. BWF helped to draft the manuscript. YPP conceived of the study, participated in its design and coordination and helped to draft the manuscript. All authors read and approved the final manuscript.
